# A Clathrin Independent Macropinocytosis-Like Entry Mechanism Used by Bluetongue Virus-1 during Infection of BHK Cells

**DOI:** 10.1371/journal.pone.0011360

**Published:** 2010-06-29

**Authors:** Sarah Gold, Paul Monaghan, Peter Mertens, Terry Jackson

**Affiliations:** Pirbright Laboratory, Institute for Animal Health, Woking, United Kingdom; University of Minnesota, United States of America

## Abstract

Acid dependent infection of Hela and Vero cells by BTV-10 occurs from within early-endosomes following virus uptake by clathrin-mediated endocytosis (Forzan *et al.*, 2007: J Virol 81: 4819–4827). Here we report that BTV-1 infection of BHK cells is also dependent on a low endosomal pH; however, virus entry and infection were not inhibited by dominant-negative mutants of Eps15, AP180 or the ‘aa’ splice variant of dynamin-2, which were shown to inhibit clathrin-mediated endocytosis. In addition, infection was not inhibited by depletion of cellular cholesterol, which suggests that virus entry is not mediated by a lipid-raft dependent process such as caveolae-mediated endocytosis. Although virus entry and infection were not inhibited by the dominant-negative dynamin-2 mutant, entry was inhibited by the general dynamin inhibitor, dynasore, indicating that virus entry is dynamin dependent. During entry, BTV-1 co-localised with LAMP-1 but not with transferrin, suggesting that virus is delivered to late-endosomal compartments without first passing through early-endosomes. BTV-1 entry and infection were inhibited by EIPA and cytochalasin-D, known macropinocytosis inhibitors, and during entry virus co-localised with dextran, a known marker for macropinocytosis/fluid-phase uptake. Our results extend earlier observations with BTV-10, and show that BTV-1 can infect BHK cells via an entry mechanism that is clathrin and cholesterol-independent, but requires dynamin, and shares certain characteristics in common with macropinocytosis.

## Introduction

Virus internalisation is initiated by binding to specific receptors at the cell-surface and culminates in host-cell membrane penetration and delivery of the viral genome to the site of replication. For many viruses, membrane penetration follows virus endocytosis. Several different endocytosis pathways operate in mammalian cells which deliver cargoes to distinct intracellular locations [1], [2]. Clathrin-mediated endocytosis (CME) is the major pathway for receptor-dependent endocytosis in many cell types and is dependent on adaptor proteins, such as the AP-2 complex, the GTPase dynamin [2], [3], and several other important accessory molecules including AP180 and Eps-15 [4], [5], [6]. CME delivers cargoes to acidic endosomes; initially to early-endosomes where they are sorted for transport to either late-endosomes/lysosomes for degradation or to recycling-endosomes for recycling back to the cell surface. Several acid-activated viruses are known to exploit the low pH within vesicles derived from the clathrin pathway, to trigger capsid un-coating and/or membrane penetration [7].

Clathrin-independent endocytosis includes the caveolae pathway which is dependent on caveolin, lipid-rafts and cholesterol [1], [2]. Cargoes internalised by caveolae are delivered to neutral pH caveosomes [8], [9]. From caveosomes, cargoes can traffic to the ER or Golgi [10], [11], [12]. Like CME, caveolae-mediated uptake requires dynamin and can be inhibited by expression of dominant-negative (DN) mutants of dynamin-2 [10], [12]. A number of viruses use caveolae for infection [10], [13], [14], [15], [16] and much of our knowledge of caveolae comes from studies with simian virus-40 [10], [17], [18]. A number of caveolin-independent pathways also originate from lipid-rafts, and novel clathrin- and raft-independent processes have also been identified. These pathways have different requirements for cellular proteins such as dynamin, flotillin, small GTPases, and for cholesterol and other specific lipids [1], [2], [19], [20], [21], [22], [23], [24], [25], and can also be exploited by viruses for infection [21], [26], [27], [28], [29], [30], [31].

Macropinocytosis is the major form of endocytosis responsible for the majority of fluid-phase uptake for a number of cell types [32]. Macropinocytosis is characterised by actin-dependent reorganisations of the plasma membrane to form macropinosomes, morphologically heterogenic vesicles that lack coat structures. Macropinocytosis is constitutive in many cell types but in others requires activation by growth factors [32], [33], [34] and contributes significantly to antigen presentation by specialised antigen presenting cells [32], [34], [35]. Macropinocytosis is also exploited by a wide range of important animal pathogens for cell invasion and possibly the avoidance of immune surveillance [36].

Here we have investigated the entry route used by bluetongue virus serotype 1 (BTV-1) to infect BHK cells. *Bluetongue virus* (BTV) is the ‘type’ species of the genus *Orbivirus* within the family *Reoviridae* which includes many important pathogens for man and animals [37]. BTV exists as at least 25 serotypes [38], [39] and is the etiological agent of bluetongue, a severe and economically important haemorrhagic disease of ruminants. Bluetongue is arthropod-borne and primarily transmitted between susceptible animal hosts by certain species of *Culicoides* biting midges [40].

BTV is non-enveloped and consists of a 3 layered protein capsid (outer-capsid, core and sub-core) surrounding a genome of ten linear segments of double-stranded RNA. The outer capsid is formed from two viral proteins, VP2 and VP5, while the inner core-particle consists of a ‘core-surface’ layer composed of VP7, which surrounds a sub-core shell composed of VP3. VP2 interacts with host cell-surface receptors serving as a virus-attachment protein while VP5 is involved in host-cell membrane penetration [41], [42], [43]. The outer capsid proteins are removed during cell entry, and transcriptionally active core particles are released into the cytosol where virus replication occurs [44], [45]. However, the core-particle is also infectious in its own right demonstrating that VP7 can also mediate cell attachment and membrane penetration, possibly by a distinct mechanism [46]. Other viral encoded proteins (NS1, NS2 and NS3) are also produced in infected cells where they are involved in virus replication and release of progeny virus particles [47].

Infection by BTV is acid-activated and requires the low pH within endosomes for disassembly of the outer viral capsid and membrane penetration, and BTV particles have been described inside endosomes which have the appearance of clathrin-coated vesicles [48]. Recent studies have concluded that entry and infection of Vero and Hela cells by BTV-10 occurs via CME, with capsid disassembly and membrane penetration within early-endosomes [45]. Here we describe the use of pharmacological and DN inhibitors of endocytosis to investigate entry and infection of BHK cells by BTV-1. We found that the clathrin pathway is not the major entry route used by BTV-1 to infect BHK cells. Instead we found that the entry mechanism shares certain characteristics in common with macropinocytosis and appears to deliver virus directly to late endosomal compartments. These studies extend earlier observations and show that BTV joins an increasing number of viruses that can exploit multiple endocytosis pathways for infectious entry.

## Methods

### Cells and viruses

Baby Hamster Kidney (BHK)-21 cells (clone 13) were obtained from the European Cell Culture Collection and maintained at 37°C, 5% CO_2_, in Glasgow Minimum Essential Medium (GMEM) (Sigma) containing 10% foetal bovine serum (FBS) (Autogen Bioclear), 2 mM glutamine, 100 U/ml penicillin, 100 µg/ml streptomycin and 5% tryptose phosphate broth solution (Sigma).

The South African reference strain of BTV-1 (IAH reference number RSArrrr/01, ICTVdb isolate accession number 41010B4F) was grown on BHK cells and gradient purified according to previously published methods [49]. Purified virus was stored at 4°C in the presence of sodium-N-lauroylsarcosine (0.1%) to prevent virus aggregation and was used for all experiments [46], [49]. Viruses were diluted immediately before use thereby reducing the concentration of sodium-N-lauroylsarcosine to <0.01% which showed no cytotoxic effects.

### Antibodies and reagents

Rabbit anti-BTV/NS2 (Orab 1) and the Guinea-pig anti-BTV/VP5 (PM10) antibodies were produced at the Institute for Animal Health using recombinant NS2 and BTV-1 as immunogens respectively. The specificity of these antibodies was verified by western blotting against purified BTV-1 and a BTV-1 infected BHK cell lysate using uninfected cells as a negative control, and by showing a lack of cross-reactivity with uninfected BHK cells by confocal microscopy. Mab 9E10 (anti-c-myc) was from the Developmental Studies Hybridoma Bank (University of Iowa). The mouse monoclonal antibody (Mab 4A1) to Lysosomal Antigen -1 (LAMP-1) was from Jean Gruenberg (University of Geneva). Species specific, Alexa-Fluor conjugated secondary antibodies were from Invitrogen.

Methyl-β-cyclodextrin, filipin, cytochalasin-D, dynasore monohydrate, and 5-(N-Ethyl-N-isopropyl)-amiloride (EIPA) were from Sigma. Ammonium chloride and concanamycin-A were from Fluka. Latrunculin-A, and Alexa-Fluor labelled human transferrin, dextran and phalloidin were from Invitrogen. Stock solutions of Methyl-β-cyclodextrin, transferrin and dextran were made in GMEM, and ammonium chloride in sterile water. Stock solutions of phalloidin were made in methanol. Stock solutions of other inhibitors were made in dimethyl sulfoxide (DMSO). Where appropriate, an equivalent dilution of DMSO (or methanol) was included in the mock treatment.

### Plasmids and cell transfection

The plasmid for expression of c-myc tagged AP180C was from Harvey McMahon (MRC. Cambridge. UK). Plasmids for expression of green fluorescent protein (GFP)-tagged wt and dominant-negative (DN)-dynamin-2 (K44A) were from Mark McNiven (Mayo Clinic. Rochester. USA). Plasmids for GFP-DN-Eps15 and control GFP-Eps15 were from Alexandre Benmerah (Université Paris Descartes. Paris. France).

Cells were seeded on glass coverslips (BDH) in antibiotic-free cell-culture medium and transfected when ∼60% confluent. Cells were transfected in Optimem (Invitrogen) using a ratio of 1 µg plasmid DNA to 1 µl Lipofectamine 2000 (Invitrogen) according to the manufacturers guidelines. Cells were incubated at 37°C, 5% CO_2_ for 4 h when the transfection medium was replaced with antibiotic-free cell-culture medium. Cells were used for experiments at 12 h post-transfection. Cells expressing a transgene were identified by confocal microscopy. Transfection efficiencies (determined by GFP or c-myc expression) ranged from 30–43%.

### Virus entry, infection and inhibitor assays

For entry experiments with transfected cells, BTV-1 (13 µg/ml in GMEM) was bound to the cells at 4°C for 40 minutes. Unbound virus was removed by washing and the cells placed at 37°C (in GMEM) to initiate entry. Where specified, inhibitors were added to the cells for 0.5 h before the virus binding step and remained present throughout the assay. All entry experiments were ended at the times indicated by the addition of 4% paraformaldehyde (PFM), with the exception of those involving transferrin which were ended using 4% PFM with 0.25% glutaraldehyde. Input virus was detected using the anti-VP5 antibody, PM10. For quantification of virus uptake, cells with cytoplasmic labelling for BTV were scored as positive. In all experiments, ∼94% of the control cells showed virus uptake.

For infections with transfected cells, the cells were inoculated with BTV-1 at a multiplicity of infection (m.o.i.) of 1 infectious virus particle per cell, in serum-free medium for 1 h at 37°C. Unbound virus was removed by washing with GMEM and the cells returned to 37°C in cell culture medium with reduced (2%) FBS.

Inhibitors (Methyl-β-cyclodextrin, cytochalasin-D or latrunculin-A) were added to cells for 0.5 h before infection. The cells were then inoculated with BTV-1 (as described above) also in the presence of an inhibitor. Mock-treated cells were infected in the absence of an inhibitor. After the 1 h incubation with virus, the cells were washed with GMEM to remove unbound virus and returned to 37°C in cell culture medium with reduced (2%) FBS, and supplemented further with 25 mM ammonium chloride. To control for any effects on intracellular virus replication the inhibitors were also added to other cells immediately after the virus inoculums were removed and coincident with the addition of ammonium chloride.

In the experiment shown in figure 2, BTV-1 (m.o.i. = 1) was bound to the cells at 4°C for 40 minutes. The cells were then washed to remove unbound virus and placed at 37°C (in GMEM) to initiate infection. This allowed the addition of inhibitor to be synchronized with the start of infection. Concanamycin-A was added to the cells at the noted times. When used as a pre-treatment, concanamycin-A was added for 0.5 h at 37°C before the addition of virus and remained present only during the virus binding step. Concanamycin-A was also added to other cells, either coincident with the start of infection (i.e. on shifting the cells to 37°C) or at various times after infection was initiated.

All infection experiments were ended after 12 h by the addition of cold 4% PFM for 40 minutes. Infected cells were identified using confocal microscopy and the anti-NS2 antibody, Orab1. For quantification of infection, cells with cytoplasmic labelling for BTV NS2 were scored as positive.

### Endocytosis of Alexa-Fluor labelled ligands

The ability of cells to internalize through the clathrin pathway was determined by observing transferrin uptake by confocal microscopy. Cells were serum starved for 0.5 h prior to the addition of Alexa-568 labelled transferrin (25 µg/ml) in GMEM at 37°C for the times indicated in the results. For quantification of transferrin uptake, cells with cytoplasmic labelling for Alexa-568 transferrin were scored as positive. Alexa-568 labelled dextran (5 mg/ml) was added to the cells as described for transferrin. In all experiments, >98% of the control cells showed transferrin or dextran uptake.

### Immunofluorescence confocal microscopy

After cell fixation with PFM, the cells were washed with PBS pH 7.5 (Sigma) and permeablized with 0.1% Triton X-100 (Sigma) in Phosphate Buffered Saline (PBS) for 20 minutes. When using Mab 4A1 to identify LAMP-1 positive compartments, the cells were permeablized with 0.5% Saponin (in place of Triton X-100) for 15 minutes and 0.1% Saponin was included in all subsequent steps. Non-specific binding sites were blocked with block buffer (100 ml Tris-buffered saline supplemented with 1 mM CaCl_2_, 0.5 mM MgCl_2_, 10% Normal Goat Serum (Harlan Lab Sera) and 1% Teleostean Gelatin [Sigma]) for 0.5 h. The cells were washed with PBS and incubated with primary antibodies in block buffer for 1 h, washed again with PBS, and incubated with the appropriate species specific, Alexa-Fluor conjugated secondary antibody (also in block buffer) for 1 h. The cells were washed and the nuclei labelled with 4′, 6′-Diamidino-2-Phenylindole (DAPI) (Sigma) in H_2_O for 10 minutes and then washed with H_2_O. The coverslips were mounted in Vectashield mounting medium for fluorescence (Vector Laboratories) and sealed with clear nail varnish.

Transfected cells expressing dynamin or Eps15 constructs were identified by the GFP fusion tag. Transfected cells expressing AP180C were identified using the anti-c-*myc* antibody (9E10) and a goat, anti-mouse Alexa-488 conjugated secondary antibody. LAMP-1 positive compartments were labelled using Mab 4A1. All data were collected sequentially to eliminate cross talk between fluorescent dyes on a Leica SP2 Confocal Scanning Laser Microscope. For experiments that involved dual labelling of virus and cellular compartments, the specificity of the secondary antibodies for the target primary antibody was confirmed by showing a lack of cross-reactivity with non-target primary antibodies. The data are shown as single optical sections through the middle of the cell.

### Labelling of cellular cholesterol and actin filaments

Cellular cholesterol was visualised at 430 nm immediately after labelling with 125 µg/ml filipin diluted in PBS for 15 minutes at room temperature [50]. To label for actin filaments, cells were fixed and permeabilized (as above) and incubated with Alexa-488 labelled phalloidin diluted in block buffer for 10 minutes at room temperature.

### Data analysis

For experiments with DN-dynamin-2 or DN-Eps15, uptake of transferrin and virus was normalised to the results obtained for cells expressing the control (wt dynamin-2 or the Eps15 control respectively). Over-expression of wt AP180 inhibits CME and cannot be used as a control for AP180C [6]. Therefore, for experiments with AP180C, transferrin or virus uptake was normalised to the results obtained for cells of the non-expressing population (i.e. the non-green cells). Similarly, for DN-dynamin-2 or AP180C, infection was normalised to the level of infection of cells expressing wt dynamin-2 or cells of the non-expressing population, respectively. The level of infection of inhibitor-treated cells was normalised to the levels of infection of the mock-treated controls. Student's *t* test was used to determine statistical significance.

## Results

### Infection of BHK cells by BTV-1 is clathrin- and cholesterol-independent, but requires active endosomal acidification

**Figure 1:**
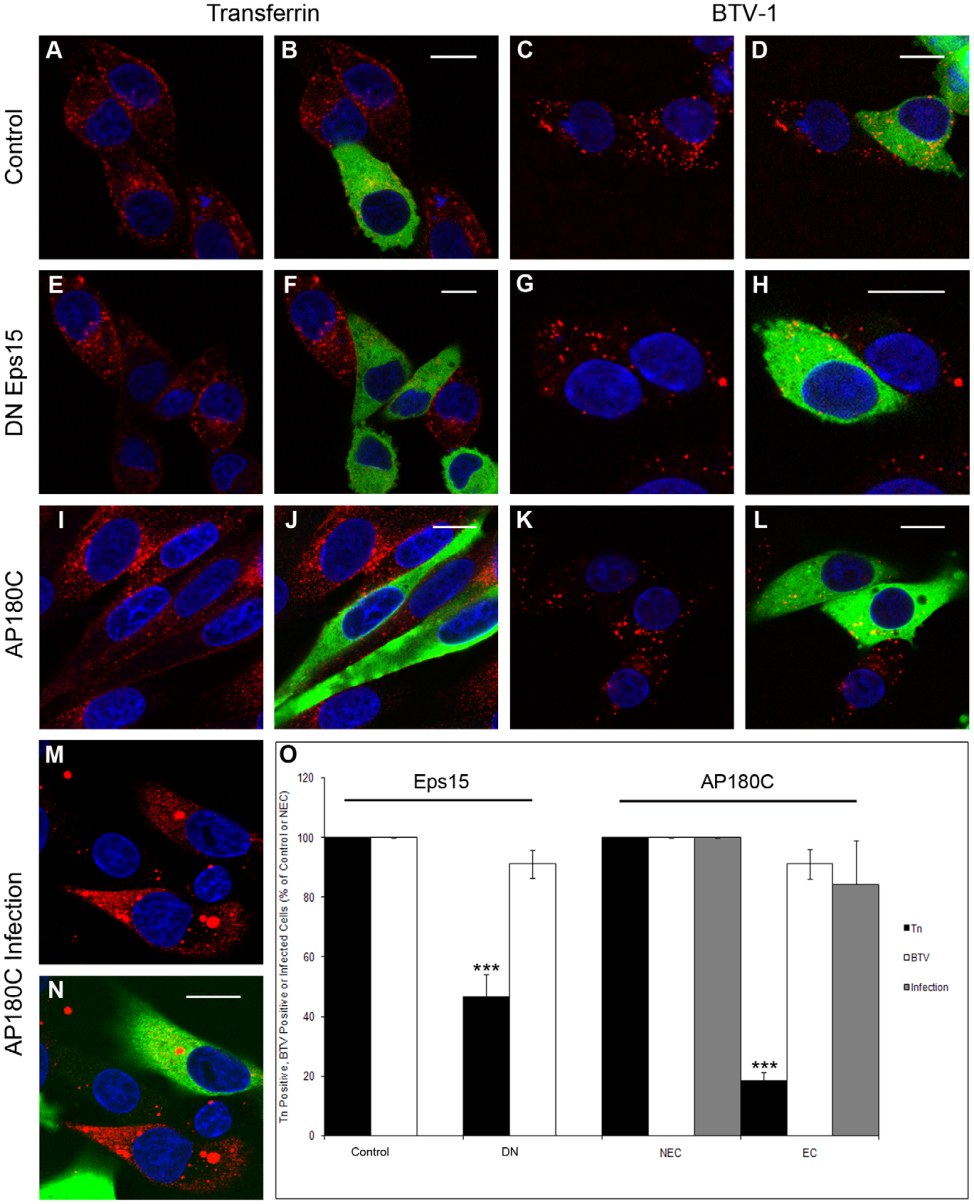
Inhibition of clathrin-mediated endocytosis does not block BTV-1 uptake or infection. BHK cells were transfected to express GFP-Eps15 control (A–D), GFP-DN-Eps15 (E–H) or AP180C-c-myc (I–N). Cells expressing a transgene are shown in green. The transfected cells were incubated with Alexa-568 transferrin or with BTV-1 (shown as red; see Methods). Panels (A), (E) and (I) show transferrin uptake, whereas panels (B), (F) and (J) show merged images for transferrin and transgene expression for the same cells as in panels A, E and I. Panels (C), (G) and (K) show uptake of BTV-1, whereas panels (D), (H) and (L) show merged images for virus labelling and transgene expression for the same cells as in panels C, G and K. Alternatively, AP180C transfected cells were infected with BTV-1 (m.o.i. = 1). At the end of the infection the cells were processed for confocal microscopy using Orab1 (anti-NS2) to identify infected cells (shown in red on Panels M and N). Panel (M) shows infected cells whereas panel (N) show a merged image for infection and AP180C expression for the same cells as in panel M. The cell nuclei are shown in blue. Scale bar  = 10 µm. Panel (O): Cells were scored positive for transferrin or BTV-1 uptake, or for infection as described in the methods. For AP180C, both the expressing and the non-expressing cell populations were or scored for transferrin uptake (total cells counted for each population >900; *n* = 6 experiments), for BTV uptake (>500 cells; *n* = 4) and for infection (>300; *n* = 3). The level of transferrin or BTV-1 uptake, or infection by the cells expressing AP180C (EC) was normalised to the level of uptake or infection by the cells of the non-expressing population (NEC). For Eps15 experiments, cells were scored for transferrin (>280 cells; *n* = 4) or BTV-1 uptake (>280 cells; *n* = 4) and level of uptake by the cells expressing DN-Eps15 (DN) was normalised to the results for the cells expressing the Esp15 control. The mean ± SD is shown for the independent experiments (*P* values: *** <0.001).

It has been reported that BTV-10 infects Hela and Vero cells using CME for entry [45]. Here we have investigated the involvement of the clathrin pathway in BTV-1 entry and infection of BHK cells using deletion mutants of AP180 (AP180C) and Eps15 (Eps15-Ed95/295; from here known as DN-Esp15) which act as DN inhibitors of CME and block uptake of transferrin [5], [6], [51]. BHK cells were transiently transfected to express AP180C-c-myc, GFP-DN-Eps15, or a control GFP-Eps15 (D3Δ2; from here known as ‘Esp15 control’) that does not interfere with the clathrin pathway [5], [51]. At 12 h post-transfection, the cells were incubated with Alexa-568 labelled transferrin for 15 minutes or with BTV-1 for 0.5 h. The cells were then fixed and processed for confocal microscopy (Fig. 1). Virus was detected using PM10 (anti-VP5) and an Alexa-568 conjugated secondary antibody. Cells expressing Eps15 proteins or AP180C were identified by the GFP or c-myc fusion tags respectively (see methods) and are shown in green on figure 1. Cells were scored for transferrin or BTV uptake as described in the methods. For each DN protein, uptake of transferrin or BTV was normalised to the results obtained for the controls (see methods). In cells expressing GFP-DN-Eps15 the frequency of transferrin uptake was reduced by 53% when compared to cells expressing the Eps15 control (Fig. 1A, B, E, F and O) indicating a block to clathrin-mediated endocytosis. Similarly, the frequency of transferrin uptake was reduced by 82% in cells expressing AP180C when compared to cells of the non-expressing population (Fig. 1I, J and O). In contrast, expression of DN-Eps15 (Fig. 1C, D, G, H and O) or AP180C (Fig. 1K, L and O) did not significantly inhibit virus uptake. In virtually all cells expressing the DN proteins the pattern of virus labelling was indistinguishable from cells expressing the Esp15 control or cells that were not expressing a transgene. These results show that under conditions where clathrin-mediated uptake of transferrin is inhibited BTV-1 is taken up normally by BHK cells.

The above experiments required using a relatively high BTV-1 concentration (13 µg/ml), to allow virus detection during entry. This raises the possibility that the virus may have been forced to enter the cells via an endocytic pathway that is not normally used, or is non-productive for infection. Therefore we also investigated the effect of AP180C expression on BTV-1 infection at a low m.o.i., to encourage virus uptake by the most efficient route. At 12 h post-transfection, the cells were incubated with BTV-1 (m.o.i. = 1) for 1 h at 37°C. The cells were then washed to remove excess virus and infection continued for a further 11 h when the cells were fixed and processed for confocal microscopy. Cells expressing AP180C were identified using expression of the c-myc tag as described above. Infected cells were identified using a rabbit antibody (Orab1) that binds the viral NS2 protein (a marker of virus replication) and an Alexa-568 conjugated secondary antibody. Intense areas of NS2 labelling were detected within infected cells (Fig. 1M and N). Cells expressing AP180C were scored for infection (indicated by labelling for NS2) and the results normalised to the results obtained for the non-expressing cell population. In the cells expressing AP180C the frequency of infection was similar to that of the non-expressing cell population, indicating that AP180C does not inhibit infection (Fig. 1O). These results show that virus uptake by cells expressing AP180C leads to infection and support the conclusion that CME is not the major pathway used by BTV-1 for entry into BHK cells.

**Figure 2:**
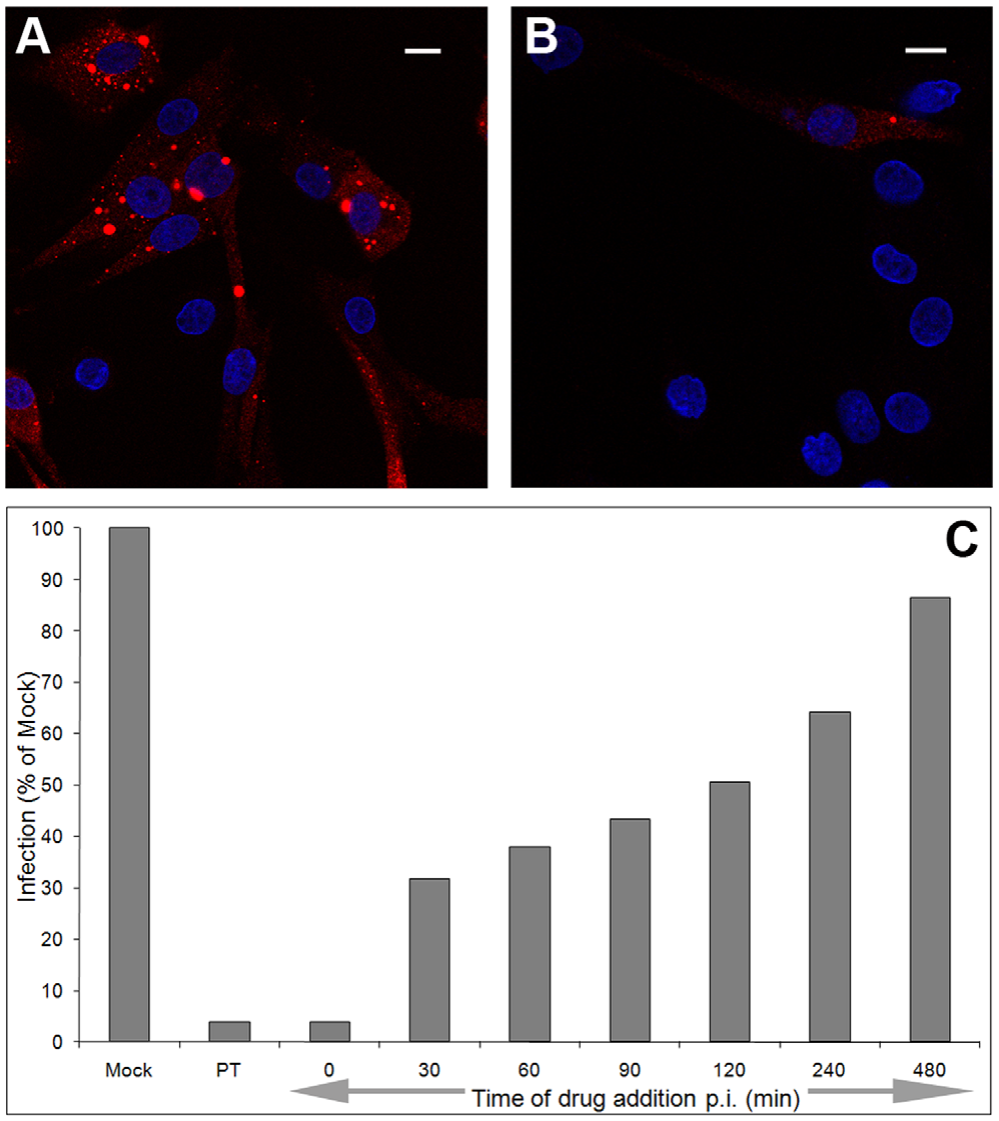
Active endosomal acidification is required for BTV infection. BHK cells were mock-treated or pre-treated (PT) with 12 nM concanamycin-A, and then infected with BTV-1 (m.o.i. = 1). Alternatively, concanamycin-A was added to the cells coincident with the start of infection (Time 0 on panel C) or at the noted times after infection was initiated. At the end of the infection the cells were processed for confocal microscopy counting at least 700 cells per time point. Infected cells were identified using Orab1 (anti-NS2) and are shown in red. Panel (A) shows mock treated cells infected with BTV-1. Panel (B) shows infected cells that had been pre-treated with drug. The cell nuclei are shown in blue. Scale bar  = 10 µm. Panel (C): The level of infection of the drug treated cells was normalised to the levels of infection of the mock treated cells. The mean of duplicate samples for one experiment of two is shown, each giving similar results.

BTV infection is known to be dependent on the low pH within endosomes [45]. Concanamycin-A is a potent and specific inhibitor of the vacuolar proton ATPase and is commonly used to raise endosomal pH [52]. Pre-treatment of BHK cells with concanamycin-A inhibited BTV-1 infection (Fig. 2A and 2B) confirming the requirement for active endosomal acidification. Similarly, when concanamycin-A was added coincident with the start of infection (i.e. at time 0 on figure 2C) a strong inhibitory effect was also seen. When added after the start of infection, concanamycin-A also had an inhibitory effect on infection. When added 2 h after infection was initiated, the frequency of infection was reduced by ∼50% when compared to mock treated cells (Fig. 2C). Near identical results were obtained using ammonium chloride in place of concanamycin-A (data not shown). These results suggest that BTV-1 is delivered to acidic endosomes much slower than expected for uptake by CME [53].

Raising endosomal pH may prevent receptor recycling, which could deplete the cell surfaces of attachment receptors and thereby reduce infection. However, the pattern of virus uptake by concanamycin-A treated cells (detected at 0.5 h post-uptake by confocal microscopy using the PM10 antibody) was indistinguishable from mock-treated cells (data not shown), indicating that the inhibitory effect of concanamycin-A on infection was not due to a failure of the virus to be internalised.

**Figure 3:**
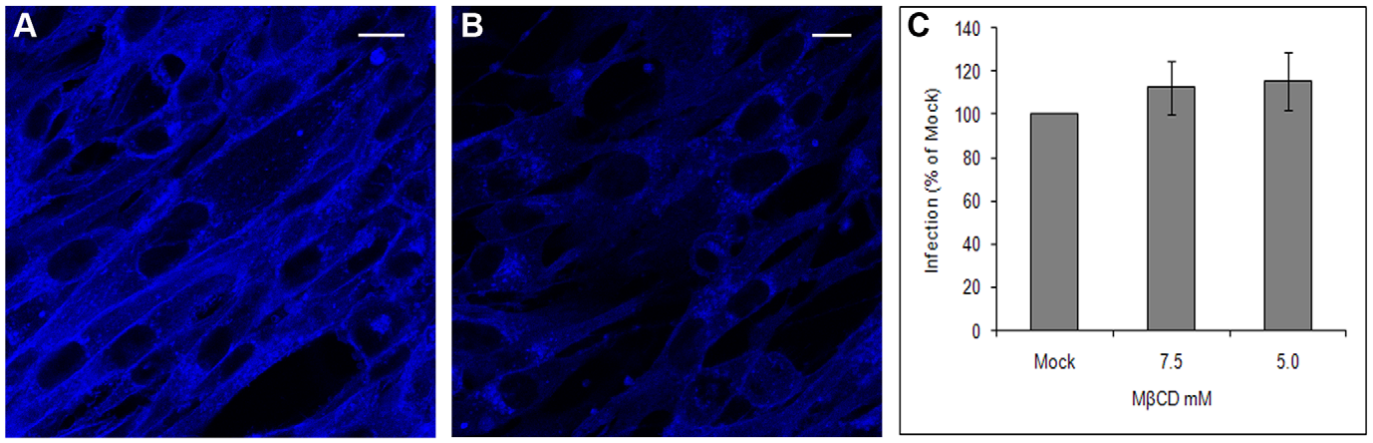
Selective cholesterol depletion from the plasma membrane does not inhibit BTV-1 infection. BHK cells were mock-treated (A) or pre-treated with 7.5 mM MβCD (B) and then incubated with filipin and viewed by confocal microscopy (see Methods). Blue fluorescence represents filipin binding to cholesterol. Scale bar  = 10 µm. Panel (C): MβCD and mock-treated cells were infected with BTV-1 (m.o.i. = 1). At the end of the infection the cells were labelled using Orab1 (anti-NS2) and scored for infection by confocal microscopy counting at least 500 cells for both the mock-and drug-treated cells. The level of infection of the drug-treated cells was normalised to the level of infection of the mock-treated cells. The mean ± SD for triplicate samples of one experiment representative of two is shown, each giving similar results.

It has recently been shown that cargo internalised via caveolae can be delivered to acidic early-endosomes [17]. Caveolae-mediated endocytosis is dependent on the integrity of lipid-rafts, and like other raft mediated endocytic pathways can be inhibited by cholesterol depletion [8], [20]. The effect of cholesterol depletion on BTV-1 infection was investigated using methyl-β-cyclodextrin (MβCD) which binds and extracts cholesterol from the plasma membrane. BHK cells were mock-treated or treated with MβCD for 0.5 h at 37°C. The cells were then incubated with filipin which binds cholesterol and can be detected by fluorescence microscopy. Filipin fluorescence was detected at the plasma membrane and within the cytosol of mock-treated cells (Fig. 3A). In contrast, although fluorescence was detected within the cytosol, the intense plasma membrane fluorescence generated by filipin binding was greatly reduced by MβCD, indicating extensive and selective cholesterol depletion from the cell surfaces (Fig. 3B).

MβCD treated cells were also infected with BTV-1 (m.o.i. = 1) for 1 h in the presence of the drug (see methods). Mock-treated cells were infected in parallel. After washing the cells to remove excess virus and drug, infection was continued for a further 11 h. At the end of infection, the cells were processed for confocal microscopy using NS2 to detect infected cells. Figure 3C shows that pre-treatment of cells with MβCD did not inhibit infection when compared to mock-treated cells. These results show that the cell entry mechanism used by BTV-1 to infect BHK cells does not require cholesterol and is, therefore unlikely to be mediated by caveolae or other lipid-raft mediated endocytic pathways.

### Entry of BTV-1 into BHK cells is dynamin dependent

**Figure 4:**
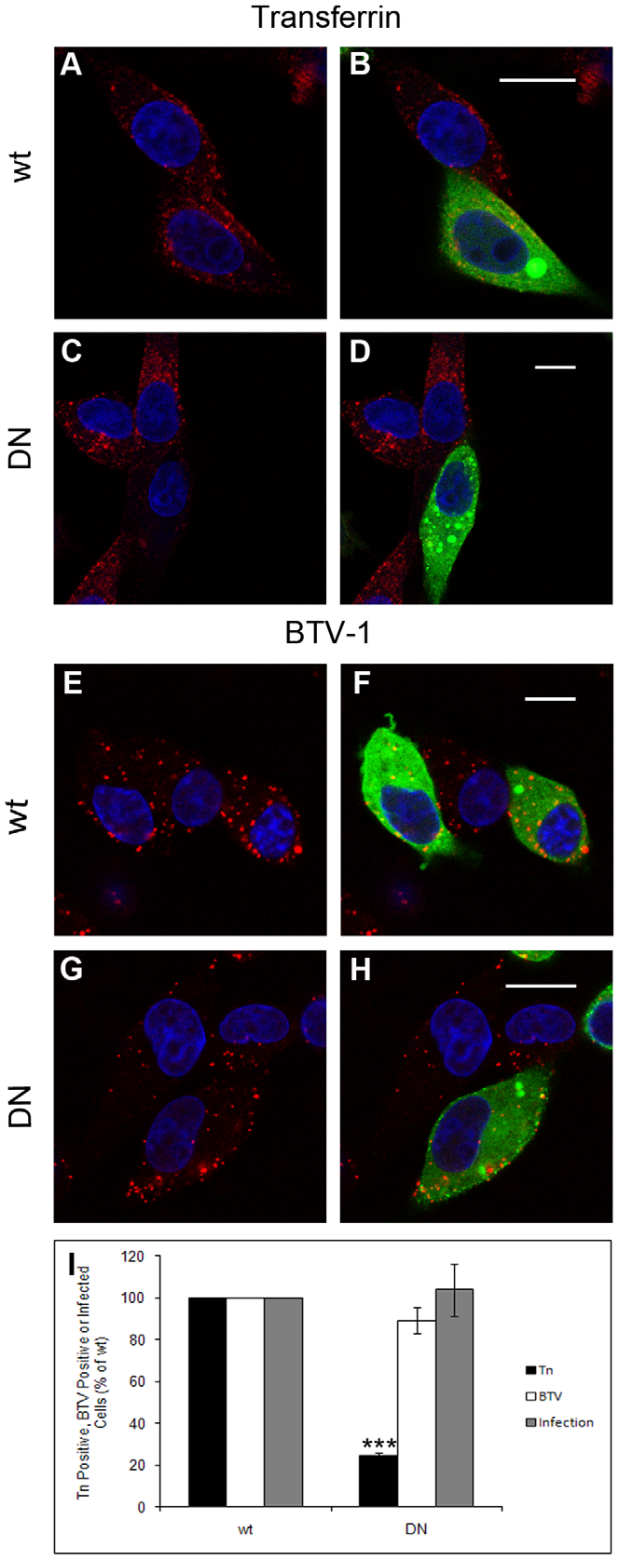
DN-dynamin-2 does not inhibit BTV-1 infection. BHK cells were transfected to express wt dynamin-2 (Panels A, B, E and F) or DN-dynamin-2 (panels C, D, G and H). Cells expressing the transgene are shown in green. The transfected cells were incubated with Alexa-568 transferrin or with BTV-1 (shown as red). Panels (A) and (C) show transferrin uptake, whereas panels (B) and (D) show merged images for transferrin and transgene expression for the same cells as in panels A and C. Panels (E) and (G) show uptake of BTV-1, whereas panels (F) and (H) show merged images for virus and transgene expression for the same cells as in panels E and G. The cell nuclei are shown in blue. Scale bar  = 10 µm. Alternatively, transfected cells were infected with BTV-1 (m.o.i. = 1). At the end of the infection the cells were labelled using Orab1 (anti-NS2) and scored for infection by confocal microscopy. Panel (I): Cells were scored positive for transferrin or BTV-1 uptake, or for infection as described in the methods counting at least 220 cells (*n* = 3 experiments) for each condition. The level of transferrin or BTV-1 uptake, or infection by the cells expressing DN-dynamin-2 (DN) was normalised to the level of uptake or infection by the cells expressing wt dynamin-2 (wt). The mean ± SD is shown for three independent experiments (*P* values: *** <0.001).

The involvement of dynamin in BTV-1 entry and infection was investigated using a DN mutant of the ‘aa’ splice variant of dynamin-2 [3], [54],[55]. BHK cells were transiently transfected to express GFP-wt ‘aa’ dynamin-2, or GFP-DN ‘aa’ dynamin-2. At 12 h post-transfection, the cells were incubated with Alexa-568 labelled transferrin for 15 minutes or with BTV-1 for 0.5 h. The cells were then fixed and processed for confocal microscopy (Fig. 4). Virus was detected using PM10 (anti-VP5) and an Alexa-568 conjugated secondary antibody. Cells expressing wt or DN-dynamin-2 were identified by the GFP fusion tag and are shown in green. The cells expressing GFP-DN-dynamin-2 showed transferrin uptake at a lower frequency than cells expressing the wt construct (Fig. 4A-D and 4I) indicating a block to clathrin-mediated endocytosis. In contrast, BTV-1 was taken up normally by cells expressing DN-dynamin-2 (Fig. 4E–H and 4I).

At 12 h post-transfection, cells were also infected with BTV-1 (m.o.i. = 1) for 1 h at 37°C. The cells were then washed to remove excess virus and infection continued for a further 11 h when the cells were fixed and processed for confocal microscopy. Infected cells were identified using a rabbit antibody (Orab1) as described above. The frequency of infection of the cells expressing DN-dynamin-2 was normalised to the level of infection of the cells expressing the wt protein. Figure 4I shows that expression of DN-dynamin-2 does not inhibit BTV-1 infection. These results are consistent with those obtained using DN-Esp15 and AP180C and support our conclusion that BTV-1 can enter BHK cells using a clathrin-independent pathway. Furthermore, as the ‘aa’ splice variant of DN-dynamin-2 also inhibits caveolae-mediated endocytosis, the above results also support our conclusion that caveolae are unlikely to be required for virus entry and infection.

**Figure 5:**
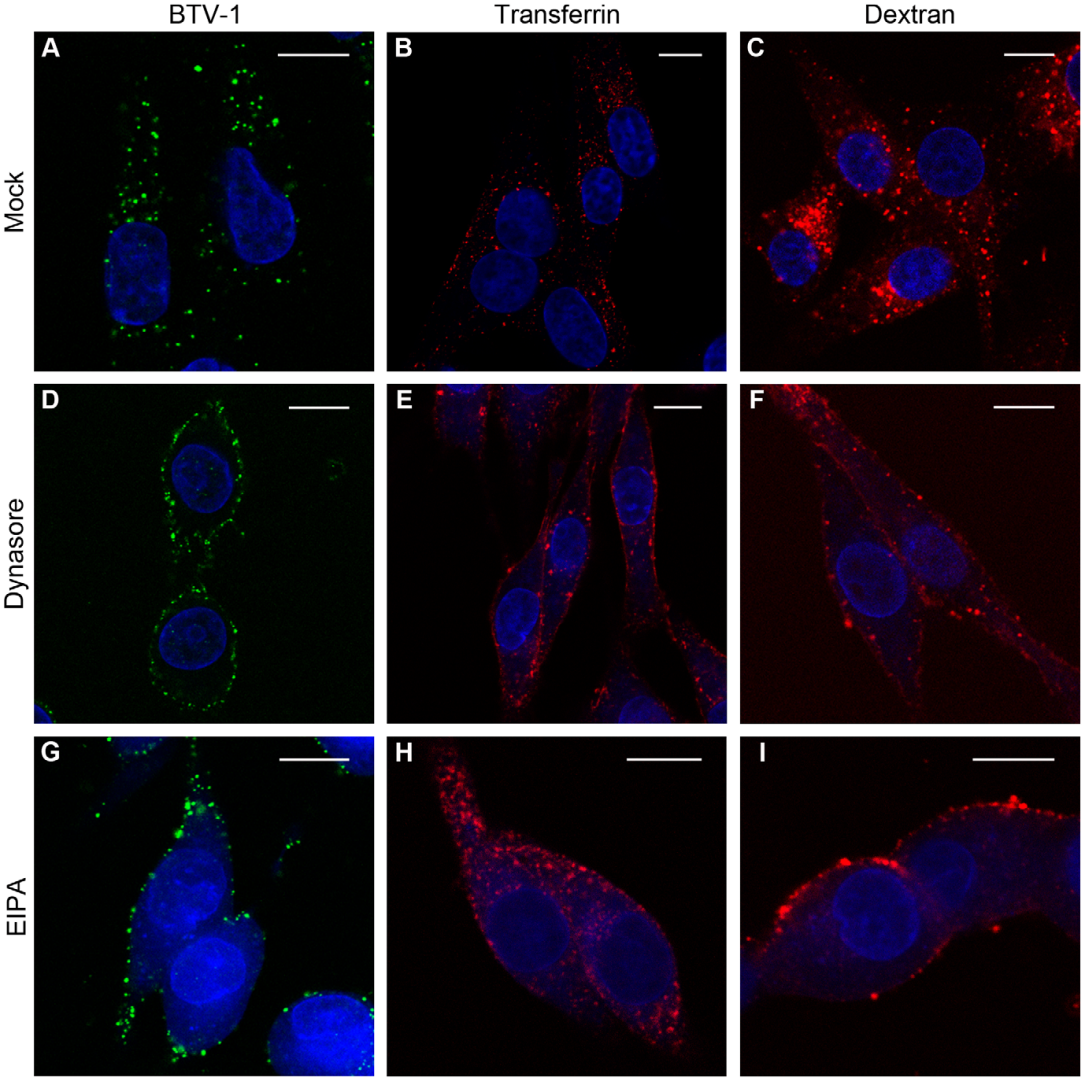
EIPA and dynasore inhibit BTV-1 endocytosis. BHK cells were mock-treated (A–C) or pre-treated with 100 µM dynasore (D–F), or 100 µM EIPA (G–I) and allowed to internalise BTV-1 (Panels A, D and G) or Alexa-568 labelled dextran (Panels C, F and I) for 0.5 h, or Alexa-568 labelled transferrin (Panels B, E and H) for 15 minutes, and processed for confocal microscopy using PM10 to detect input virus (shown as green). The cell nuclei are shown in blue. Note: EIPA treatment results in a faint blue fluorescence throughout the cytosol. Scale bar  = 10 µm.

To further investigate the role of dynamin in BTV-1 infection, BHK cells were treated with the general dynamin inhibitor, dynasore [56]. Mock-treated and dynasore-treated cells were incubated with BTV-1, Alexa-568 labelled transferrin or Alexa-568 labelled dextran. Figure 5A-F shows representative cells and that dynasore effectively inhibited uptake of all three ligands. Virtually all of the drug treated cells (*n*>300 cells per ligand) showed no uptake of virus, transferrin or dextran.

We also investigated the effects of dynasore on BTV-1 infection. However, these experiments were inconclusive as ∼6 h after the drug was removed, the cells detached from the coverslips indicating a delayed cytotoxic effect. Nevertheless, the observation that dynasore inhibits entry of BTV-1 into BHK cells shows that the entry mechanism is dynamin dependent.

### BTV-1 entry into BHK cells shares characteristics with macropinocytosis

**Figure 6:**
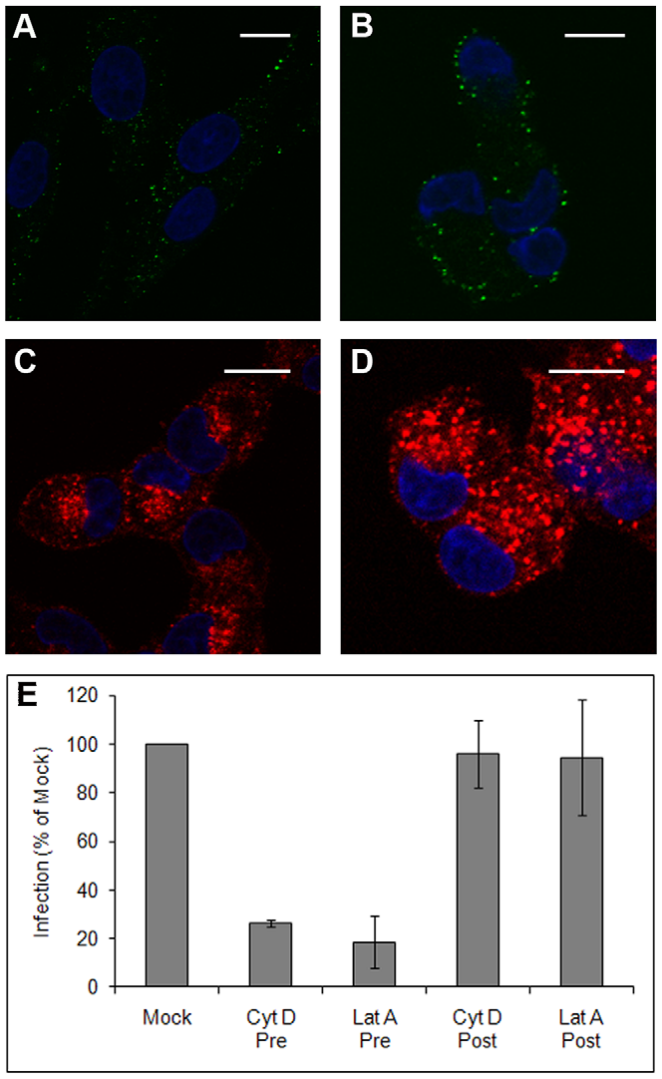
Actin disruption inhibits BTV-1 infection. BHK cells were treated with 2 µM cytochalasin-D and allowed to internalise 568-Aexa labelled transferrin (shown as red) for 15 minutes (Panel C), or 568-Alexa labelled dextran (shown as red) (Panel D) or BTV-1 (shown as green) (Panels B) for 0.5 h. Panel A show virus uptake by mock-treated cells. The cell nuclei are shown in blue. Scale bar  = 10 µm. BHK cells were pre-treated (Pre) with 2 µM cytochalasin-D or 1.5 µM latrunculin-A, or mock-treated and then infected with BTV-1 (m.o.i. = 1). The drugs were also added to other cells after the start of infection (Post) (see Methods). At the end of the infection the cells were labelled using Orab1 (anti-NS2) and scored for infection by confocal microscopy counting at least 300 cells per condition. The level of infection of the drug-treated cells was normalised to the level of infection of the mock-treated cells. The mean ± SD for triplicate samples of one experiment representative of two is shown, each giving similar results.

A number of endocytic processes are dependent on functional actin dynamics [2]. Therefore we determined if BTV-1 entry into BHK cells requires actin using cytochalasin-D which disrupts actin filaments. Cells were mock-treated, or treated with the drug and successful disruption of actin filaments confirmed by confocal microscopy after labelling the cells with Alexa-488 labelled phalloidin (data not shown). Mock or drug-treated cells were allowed to internalise BTV-1 (for 0.5 h) in the presence of the drug. The cells were then fixed and processed for confocal microscopy using PM10 to detect input virus as described above. Figure 6A and 6B show representative cells and that cytochalasin-D effectively blocked entry of BTV. Virtually all of the drug-treated cells (*n*>200 cells) showed a pattern of virus labelling as shown on the figure. In contrast, figure 6C and 6D show that cytochalasin-D did not inhibit uptake of 568-Aexa labelled transferrin, or 568-Alexa labelled dextran. These results show that BTV-1 uptake by BHK cells is actin-dependent and confirms that BTV-1 and transferrin enter cells via different mechanisms.

Cytochalasin-D treated cells were also infected with BTV-1 for 1 h in the presence of the drug (see methods). Mock-treated cells were infected in parallel. After the 1 h incubation with virus the cells were washed to remove excess unbound virus and inhibitor, and infection continued for a further 11 h. At the end of infection the cells were processed for confocal microscopy using NS2 to detect infected cells. The effects of cytochalasin-D on actin filaments are reversible and it is possible that following drug wash-out, virus that remained bound to the outsides of the cells (as a result of actin disruption) may cause an infection. Therefore ammonium chloride was added to the culture medium immediately after the virus inoculums and cytochalasin-D was removed. Ammonium chloride is a membrane permeable weak base that rapidly raises endosomal pH [57] and was added to prevent further infection events by virus that have not yet entered the cell and undergone acid-induced membrane penetration. Under these conditions infection can only occur during the period when cytochalasin-D was present. To control for possible effects on intracellular virus replication, cytochalasin-D was also added to other cells immediately after the virus inoculums were removed and co-incident with the addition of ammonium chloride. Under these conditions, cytochalasin-D can only have an inhibitory effect on infection by interfering with post-entry virus replication. Figure 6E shows that pre-treatment of BHK cells with cytochalasin-D inhibited BTV-1 infection. In contrast, when added after the virus internalisation step no inhibitory effect was seen (Fig. 6E). Similar results were obtained using latrunculin-A in place of cytochalasin-D (Fig. 6E). These results confirm that BTV-1 infectious entry into BHK cells is actin dependent.

Macropinocytosis is actin dependent and is increasingly recognised as an important cell entry route for a number of viruses [36]. Macropinocytosis is also dependent on the amiloride-sensitive Na^+^/H^+^ exchangers [36] and is inhibited by 5-(N-ethyl-N-isopropyl)-amiloride (EIPA). EIPA has previously been shown to inhibit macropinocytosis without affecting other endocytic pathways such as CME [58]. Figure 5 shows representative cells and that EIPA effectively inhibited uptake of dextran (Fig. 5I) and BTV-1 (Fig. 5G). Virtually all of the drug treated cells showed no uptake of dextran or virus (*n*>300 cells counted for each ligand). In contrast, EIPA did not inhibit uptake of transferrin (Fig. 5H) as virtually all cells showed transferrin uptake; (*n*>300 cells counted). These results also suggest that BTV-1 entry may be mediated by macropinocytosis or a macropinocytosis-like mechanism. In addition, as EIPA blocked uptake of BTV-1 but not transferrin, these observations strengthen our conclusion that BTV-1 entry is not mediated by the clathrin pathway.

**Figure 7:**
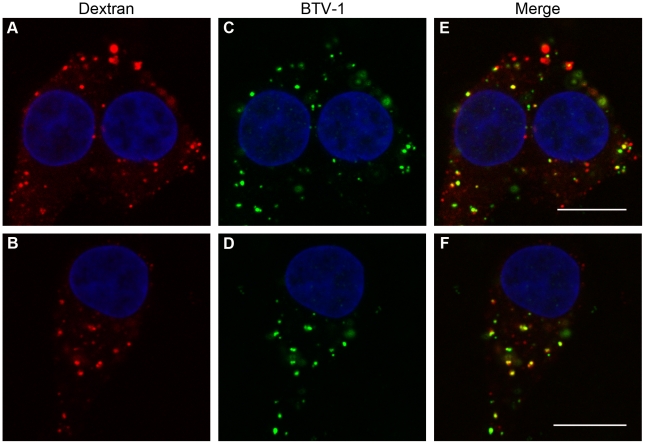
BTV-1 is co-localised with dextran during cell-entry. BTV-1 was pre-bound to BHK cells and then internalised in the presence of Alexa-568 dextran (red) for 0.5 h. The cells were then processed for confocal microscopy using PM10 to detect input virus (green). Panels (A) and (B) shows dextran uptake for representative cells. Panels (C) and (D) shows virus uptake for the same cells as in panels A and B respectively. Panels (E) and (F) show merged images for the same cells. Yellow indicates co-localisation. The cell nuclei are shown in blue. Scale bar  = 10 µm.

Although commonly used as a marker to indicate uptake by macropinocytosis, dextran is likely to enter cells by other pathways that participate in fluid-phase uptake. Certainly, our results with cytochalasin-D show that dextran can enter BHK cells using an actin-independent pathway, as although actin filaments were disrupted by cytochalasin-D, dextran uptake appeared to be unaffected (see Fig. 6D). Nevertheless, figure 7 shows that at 0.5 h and 2 h post uptake, BTV-1 was co-localised with co-internalised Alexa-568 labelled dextran, which also suggest that BTV-1 may use macropinocytosis or a macropinocytosis-like mechanism to enter BHK cells.

### BTV-1 is delivered to Lamp-1 positive endosomes during entry

The results described above show that BTV-1 infection of BHK cells occurs via a clathrin-independent entry mechanism that shares certain characteristics with macropinocytosis. Macropinosomes can fuse with lysosomes or recycle their contents back to the extracellular space, most likely via early- and recycling-endosomes [32], [59], [60]. Therefore, we investigated whether BTV-1 was delivered to these compartments during entry. BTV-1 was internalised for 15, 30, 60, 90 and 120 minutes before the cells were fixed and processed for confocal microscopy using PM10 (anti-VP5) to detect internalised virus. Early- and recycling-endosomes were labelled by incubating the cells with Alexa-568 labelled transferrin during the final 15 minutes of virus uptake (i.e. for 15 minutes before the cells were fixed). Alternatively, other cells were double labelled for virus (as above) and LAMP-1 (a marker for late-endosomes and lysosomes) using Mab 4A1.

**Figure 8:**
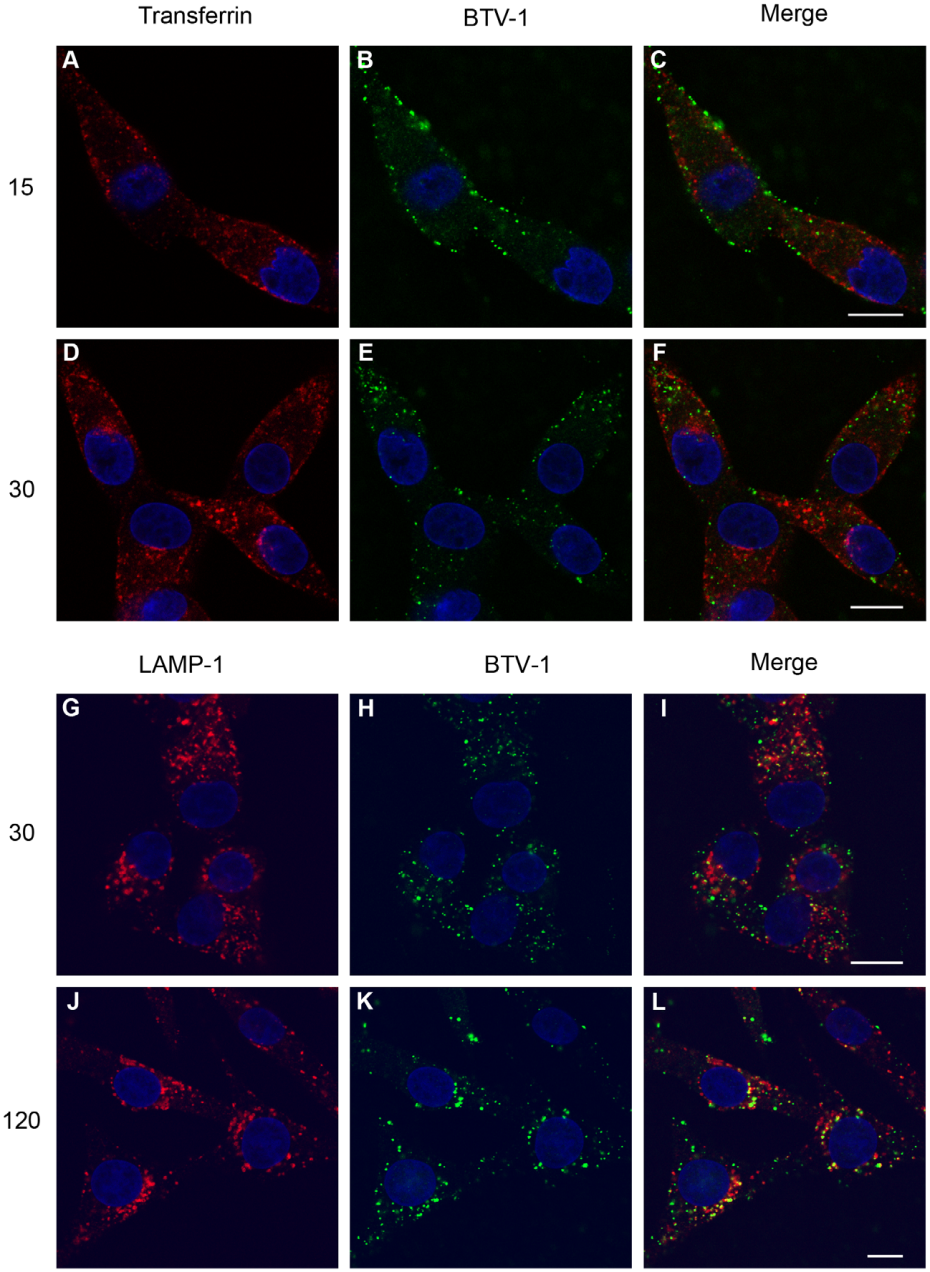
During entry BTV-1 is delivered directly to LAMP-1 positive compartments. BTV-1 was pre-bound to BHK cells and then internalised for 15, 30, or 120 minutes and the cells processed for confocal microscopy using PM10 to detect input virus (green). Panels A–F: Alexa-568 labelled transferrin was added to the cells during the final 15 minutes of virus uptake, to label early and re-cycling endosomes. Panels (A) and (D) show transferrin uptake. Panel (B) and (E) show virus uptake. Panel (C) and (F) show merged images for the same cells. Alternatively, cells were double labelled for BTV-1 and LAMP-1. Panels (G) and (J) shows labelling for LAMP-1. Panel (H) and (K) show virus uptake. Panel (I) and (L) show merged images for the same cells. Yellow indicate areas of co-localisation. The cell nuclei are shown as blue. Scale bar  = 10 µm.

Figure 8, shows representative cells from these experiments. After 15 or 30 minutes of virus uptake, little or no virus co-localisation with transferrin was observed (Fig. 8A–F). Similarly, at the other times investigated, virus co-localisation with transferrin was not observed (data not shown). These results show that during the first 2 h of entry, BTV-1 does not appear to enter early- or re-cycling-endosomes, supporting the conclusion that CME is not required for BTV-1 infection. Similarly, at 15 minutes post-uptake, virus was not co-localised with LAMP-1 (data not shown). In contrast, at 0.5 h and 2 h post-entry BTV-1 was observed co-localised with LAMP -1 (Fig. 8G–L). At 0.5 and 2 h post uptake, ∼34% and ∼54% of the virus was judged to be co-localised with LAMP-1. These results are consistent with an uptake mechanism that delivers BTV-1 to LAMP-1 positive compartments (late-endosomes/lysosomes) without the need to first pass through early-endosomes.

## Discussion

Infection by Bluetongue virus is acid activated and can be inhibited by raising endosomal pH. Consistent with these observations, Forzan *et al* [45] concluded that BTV-10 infection of Vero and Hela cells is mediated by CME, with capsid disassembly and membrane penetration occurring from within early-endosomes. In this report, we have confirmed that active endosomal acidification is required for BTV-1 infection of BHK cells. However, in contrast to previous studies we found that the clathrin pathway is not the major entry route used by BTV-1 to infect BHK cells. This conclusion is based on a number of observations, including; (1) although a low endosomal pH was shown to be essential for infection, the kinetics of BTV-1 delivery to acidic compartments was slower than expected for uptake by the clathrin pathway [53]; (2) during entry, BTV-1 did not co-localise with transferrin, a commonly used marker for CME; and (3) cell-entry and infection was not inhibited by expression of three different DN inhibitors of CME (AP180C, DN-Eps15 and DN-dynamin-2). Furthermore, both EIPA and cytochalasin-D were shown to inhibit uptake of BTV-1 but not uptake of transferrin indicating that BTV-1 and transferrin are internalised by different endocytic pathways.

Our observations suggest that during entry into BHK cells, BTV-1 is delivered to acidic compartments via a clathrin independent route. One possible route is uptake via caveolae as they can undergo fusion with early-endosomes [17]. However, the failure of DN-dynamin to inhibit BTV-1 entry and infection of BHK cells suggests that virus uptake is independent of caveolae. This conclusion was further supported by showing that BTV-1 infection was not inhibited by cholesterol depletion, which disrupts several lipid-raft dependent processes including caveolae-mediated endocytosis. Another clathrin-independent uptake route that leads to acidic endosomes is the CLIC/GEEC (*CL*athrin *I*ndependent *C*arriers/*G*PI-AP *E*nriched *E*ndocytic *C*ompartments) pathway [61], [62]. However, in contrast to the entry mechanism used by BTV-1 to infect BHK cells, the CLIC/GEEC pathway is highly sensitive to cholesterol depletion [62] and is, therefore, unlikely to mediate BTV-1 uptake. Recent studies have shown that vesicles derived from a number of other clathrin-independent pathways can fuse with early- or late-endosomes [22], [24], [63], [64], and that viruses can use such mechanisms for infectious entry [15], [16], [24]. For example, Lymphocytic Choriomeningitis Virus (LCMV) and Kaposi's Sarcoma associated Herpesvirus (KSHV) are internalised by clathrin-independent mechanisms that result in virus delivery to late-endosomes without first passing through early-endosomes [65], [66], [67]. However, the uptake pathway used by LCMV appears distinct from that described here for BTV-1 entry into BHK cells in several ways including that LCMV uptake and infection are not blocked by inhibitors of macropinocytosis. The entry route used by KSHV appears more similar to that described here for BTV-1 since it also shares characteristics of macropinocytosis; however, unlike the BTV-1 BHK cell entry mechanism, entry of KSHV is not inhibited by dynasore and is therefore dynamin independent.

On the basis of virus co-localisation with dextran, and the sensitivity to EIPA or actin disruption, the BTV-1 BHK cell entry route appears to be most closely related to macropinocytosis [36]. A number of viruses including vaccinia virus [68], [69], [70], coxsackievirus B3 [71], HIV [72], KSHV [66], echovirus 11 [73], [74], human rhinovirus-14 [75] and Adenovirus type 3 [76] have been shown to exploit macropinocytosis, or macropinocytosis-like endocytic pathways for cell-entry and infection. Macropinosomes can deliver their contents to late endosomal compartments (late-endosomes or lysosomes) without the need for early-endosomes [32], [59]. Similarly, the route described here for BTV-1 entry into BHK cells also appears to by-pass early-endosomes and delivers virus to late endosomal compartments. These observations suggest that infection (i.e. acid induced membrane penetration) might occur from within late-endosomes or lysosomes. However, we cannot yet be certain that these compartments are the site of infection as the intermediate steps of virus trafficking between the plasma membrane and late-endosomal compartments are not known, and it is possible that virus could be exposed to a low pH on transit.

The involvement of dynamin in macropinocytosis is not clear and studies investigating its role have given conflicting results [55], [58], [69], [70], [77], [78]. Similarly, viruses that use macropinocytosis for infection display different sensitivities to dynamin inhibition [69], [70]. Our studies showed that a DN mutant of the ‘aa’ splice variant of dynamin-2 does not inhibit BTV-1 uptake or infection of BHK cells. This DN mutant was shown to inhibit uptake of transferrin (see Fig. 4) but not dextran (data not shown), indicating that macropinocytosis/fluid-phase uptake, but not CME is active in BHK cells expressing this mutant. These observations are consistent with recent studies by Cao et al., (2007) which show that different splice variants of dynamin-2 regulate different endocytosis pathways, and that expression of a DN mutant of the ‘aa’ splice variant of dynamin-2 resulted in attenuation of transferrin, but not dextran uptake [55]. In contrast, BTV-1 uptake was inhibited by the dynamin inhibitor, dynasore. Since it is likely that dynasore inhibits all forms of dynamin, this observation suggests that the mechanism used by BTV-1 to enter BHK cells is dynamin dependent but relies on a form of dynamin that cannot be inhibited by DN mutants of the ‘aa’ splice variant of dynamin-2.

In conclusion, we have described here a pathway for BTV-1 uptake and infection of BHK cells that is independent of clathrin and cholesterol requires dynamin, appears to deliver virus directly to late-endosomes/lysosomes without first passing through early-endosomes, and shares characteristics with macropinocytosis. Recently, Forzan *et al*., [45] reported that BTV-10 infection of Vero and Hela cells is mediated by CME with acid-induced infection occurring within early-endosomes. Hence, BTV joins an increasing number of viruses that appears able to use more than one endocytic pathway to initiate infection [21], [66], [79], [80], [81], [82]. BTV infects a wide variety of cell types in its mammalian hosts including endothelial cells [37], mononuclear phagocytes [83], γδ T cells [84], dendritic cells [85], and a variety of leukocytes [37], [86]. It is likely that this broad tropism, and the ability of BTV to replicate in such evolutionary distant hosts results, in part from an ability to use multiple entry routes to initiate infection. Moreover, BTV can exist in at least three different forms that are all considered to be infectious. These include intact virus particles, infectious sub-viral particles, and virus-cores [46]. These different particle types have different surface components, and may therefore also use different entry mechanisms for infection. However, the entry route used by BTV to infect its natural target cells and the relevance of these pathways to pathogenesis in the animal and insect hosts will require further study.
